# Exploring the Experiences of Individuals Allocated to a Control Setting: Findings From a Mobile Health Smoking Cessation Trial

**DOI:** 10.2196/12139

**Published:** 2019-04-02

**Authors:** Ulrika Müssener, Catharina Linderoth, Marcus Bendtsen

**Affiliations:** 1 Department of Medical and Health Sciences Linköping University Linköping Sweden

**Keywords:** tobacco smoking, smoking cessation, students, text messaging, mobile phones, cell phone, control groups

## Abstract

**Background:**

Tobacco smoking is the primary cause of preventable premature disease and death worldwide. Evidence of the efficacy of text messaging interventions to reduce smoking behavior is well established, but there is still a need for studies targeting young people, especially because young adult smokers are less likely to seek treatment than older adults. A mobile health intervention, Nicotine Exit (NEXit), targeting smoking among university students was developed to support university students to quit smoking. Short-term effectiveness was measured through a randomized controlled trial, which found that immediately after the 12-week intervention, 26% of smokers in the intervention group had prolonged abstinence compared with 15% in the control group.

**Objective:**

The objective of this study was to explore the experience of being allocated to the control group in the NEXit smoking cessation intervention.

**Methods:**

We asked students who were allocated to the control group in the main NEXit randomized controlled trial to report their experiences. An email was sent to the participants with an electronic link to a short questionnaire. We assessed the distribution of the responses to the questionnaire by descriptive analysis. We analyzed free-text comments to 4 questions.

**Results:**

The response rate for the questionnaire was 33.8% (258/763), and we collected 143 free-text comments. Of the responders, 60.9% (157/258) experienced frustration, disappointment, and irritation about being allocated to the control group; they felt they were being denied support by having to wait for the intervention. Monthly text messages during the waiting period thanking them for taking part in the trial were perceived as negative by 72.3% (189/258), but for some the messages served as a reminder about the decision to quit smoking. Of the responders, 61.2% (158/258) chose to wait to quit smoking until they had access to the intervention, and 29.8% (77/258) decided to try to quit smoking without support. Of the respondents, 77.5% (200/258) claimed they were still smoking and had signed up or were thinking about signing up for the smoking cessation program at the time of the questionnaire.

**Conclusions:**

Most of the respondents reported negative feelings about having to wait for the support of the intervention and that they had decided to continue smoking. A similar number decided to wait to quit smoking until they had access to the intervention, and these respondents reported a high interest in the intervention. Free-text comments indicated that some control group participants believed that they had been excluded from the trial, while others were confused when asked to sign up for the intervention again.

**Trial Registration:**

ISRCTN Registry ISRCTN75766527; http://www.isrctn.com/ISRCTN75766527 (Archived by WebCite at http://www.webcitation.org/7678sUKbR)

## Introduction

### Background

Tobacco smoking is the primary cause of preventable premature disease and death worldwide. Tobacco use is estimated to kill 7 million people per year. If current trends continue, by 2030 tobacco will contribute to the deaths of more than 8 million people a year, with 80% of those deaths predicted to occur in the developing world [1]. Most smokers start in their teens and, as tobacco use increases with age, the earlier a person starts smoking, the higher their risk of becoming addicted and developing illnesses due to smoking [2]. Identifying effective interventions to help young people to quit smoking would have a major impact on population health, in both the short and the long term.

A growing body of evidence has accumulated in support of the efficacy of short message service (SMS) text messaging programs on mobile phones for health behavior change, including smoking cessation [3-6]. A Cochrane review of 12 such studies concluded that mobile interventions doubled the chances of long-term quitting compared with control groups [4]. A metareview of 13 studies on text message interventions for smoking cessation showed that cessation rates for the intervention group were 36% higher than for the control group [7]. An earlier Cochrane review of 28 trials suggested that interventions that have been shown to be effective among adults, such as motivational enhancement, could also be effective among adolescents. However, the review also found that there is insufficient evidence to recommend a specific method of intervention for young people and that more data are needed on long-term cessation [8].

Thus, the evidence for the efficacy of text messaging interventions to reduce smoking behavior is well established, but there is still a need for studies targeting young people, especially because young adult smokers are less likely to seek treatment than older adults [9]. In addition, most of the policies and smoking programs implemented in schools and universities are preventive, and thus are effective in reducing the initiation and prevalence of smoking among adolescents, rather than supporting young smokers to stop smoking [10].

### Objective

In our previous research, we have developed a mobile health intervention, Nicotine Exit (NEXit), targeting smoking among university students (ISRCTN75766527) [11,12]. We previously tested the effectiveness of the intervention in a randomized controlled trial (RCT) and reported our results [13], as well as the satisfaction and acceptability of the intervention [12]. In RCTs, the estimated effect of the intervention under study can only be understood relative to the control setting. An effect size is estimated comparing 2 groups, 1 randomly allocated to be given access to an intervention, and 1 randomly allocated to a control setting. It is natural that the intervention setting is given much focus and is often explained in detail in research papers; however, the information given to, and experiences of, the control group plays a crucial role when interpreting results. The aim of this study was to explore participants’ experience of being allocated to the control group in the NEXit smoking cessation intervention.

## Methods

### Description of the NEXit Intervention

The NEXit intervention was developed in several steps based on existing recommended smoking cessation manuals used in Sweden, previous research, and a taxonomy on behavior change techniques developed by Michie et al [14].

Elements included in the intervention were as follows: making a public declaration about quitting (ie, telling friends about the quit attempt), encouraging asking for support from family and friends, distraction techniques, and the possibility of requesting more text messages when the participant experienced strong cravings or a temporary relapse. The core program lasted for 12 weeks and consisted of 157 messages [13].

### Ethics Approval

The study was approved by the Regional Ethical Committee in Linköping, Sweden (Dnr 2014/217-31).

### Study Population and Procedure

To get information for subsequent revisions and improvements from the individual user’s perspective, we invited college and university students participating in the main NEXit RCT to give feedback after completing the 12-week intervention and after participating in the formal follow-up of the RCT [13]. We recruited the participants from all colleges and universities in Sweden except one university that participated in a pilot study. The participants came from all levels and disciplines. Recruitment of participants was completed over a 3-week period (October 23 to November 13, 2014). A total of 827 participants were allocated to the intervention group and 763 to the control group (a waiting list group that were asked to quit on their own, but were also told that they were going to be given access to the intervention after the trial). Data on the primary outcome were collected over a 4-week period from 94% of the control group. Reminders were sent over the same 4-week period. Nonresponders to the follow-up were sent up to 6 reminders by email and 3 reminders by SMS text messaging, and 10 attempts were made to telephone those who had still not responded.

After the follow-up procedure, the control group received an email with an electronic link to a short questionnaire, with 2 reminders sent 1 week apart to nonresponders.

### Questionnaire

We asked the participants in the NEXit control group 4 questions, with 3 to 4 fixed response options. The questions were about experiences of being randomly allocated to the control group, smoking behavior when not given access to the intervention, and willingness to sign up after the trial. A free-text option after each question gave the participants an opportunity to describe other factors of importance not covered by the fixed response options.

We explored experiences of being randomly allocated to a control group by 2 questions: (1) experience of having to wait for the intervention (response options: frustrating, irritating, or disappointing because I was prepared to quit smoking; positive, inspiring, or motivating because I was given a chance to reflect on my reasons to quit smoking; it did not matter; do not know); (2) perception of receiving monthly SMS text messages during the waiting time thanking them for participating in the trial (response options: good, they made me feel part of the study and reminded me that I would get support later; bad, because I did not have access to the program; did not matter to me, I did not care about the messages; do not know)*.*

We explored actions taken when being allocated to the control group by 1 question: (3) reaction when not given access to the intervention immediately (response options: I decided to try to quit smoking without help; I decided to postpone my quit attempt until I had access to the intervention; I used other support).

We explored willingness to sign up after the trial by 1 question: (4) intention to sign up after the trial (response options: yes, I still smoke and I have signed up for the intervention; yes, I still smoke and I am still thinking about signing up; no, I still smoke but do not want any help; no, I have quit smoking).

### Statistical Analysis

We performed descriptive analysis of the distribution of the responses to the 4 questions in 4 steps. In the first step, all free-text comments to each question were read by the first and second authors (UM and CL). In the second step, CL chose a variety of the most crucial free-text comments for each question. In the third step, UM verified the chosen free-text comments and added some comments relevant to the aim of the study. In the fourth step, all authors discussed all of the chosen free-text comments, from which we selected comments that captured the main content of the specific question with regard to the aim of the study.

## Results

### Participants’ Characteristics and Response Rate

The baseline characteristics of the participants were similar to those of nonparticipants concerning sex, age, and marital status (Table 1). Further, the baseline characteristics of the control group participants were similar to those of the intervention group in the main study [13] concerning sex, age, and marital status. Thus, we regarded the participants in this study as being broadly representative of the intervention group in the RCT. The overall response rate was 33.8% (258/763).

About a third (83/258, 32.2%) of the respondents provided 143 comments to the 4 questions; the other 67.8% (175/258) did not offer any additional comments. Most comments were on the question regarding how participants reacted when they did not get access to the intervention at once (42/143, 29.4%). The fewest comments (n=26/143, 18.2%) were provided for the question on willingness to sign up for the intervention after the trial. On average, approximately 35 comments were provided for each question.

We report the responses to the 4 questions, as well as citations from the free-text comments to illustrate and underline the pattern of responses to the fixed response options. The comments were translated from Swedish into English by the first author. The designation after each quotation is the code assigned to the participant.

### Experiences of Having to Wait for the Smoking Cessation Aid

Of the respondents, 60.9% (157/258) experienced frustration, disappointment, and irritation when being told that they had to wait for the novel smoking cessation aid. In the free-text comments, some participants emphasized that being allocated to the control group and thus having to wait 4 to 5 months for access evoked feelings of being denied support and decreased their motivation to stop smoking.

I felt quite dejected; to first make the decision to participate in the program, but then being denied.

Participant 212

It felt like taking a step back, and it had a demotivating effect on my choice to quit smoking.

Participant 210

However, 10.9% (28/258) reported that having to wait for support was positive and inspiring because they had the chance to reflect on their decision to quit smoking or had time to really set a goal to quit. In their comments, some described that having to wait for smoking cessation increased their chances of succeeding and that their motivation increased.

I was prepared to quit immediately, but due to the delay my subconscious has set a goal for my smoking cessation, which increases my chances.

Participant 149

Initially I felt disappointed, but then I thought that this will turn out just fine because I will get the support later; meanwhile my decision to quit smoking has really engrained itself. It feels like my motivation to quit is much more rooted than it ever has been before.

Participant 156

Of the respondents, 28.3% (73/258) stated that having to wait for the support did not matter or they did not know whether it mattered.

### Perceptions of Receiving Text Messages During the Waiting Time

All participants allocated to the control group received monthly text messages during the waiting time thanking them for taking part in the trial. Of the respondents, 72.3% (189/258) thought these messages were bad or worthless. Among those, some highlighted that the text messages were confusing because these participants did not have access to the intervention but were still being thanked for taking part.

**Table 1:** Baseline characteristics of responders and nonresponders (N=763).

Characteristics		Nonresponders (n=505), n (%)	Responders (n=258), n (%)	*P* value^a^
Female		332 (65.7)	190 (73.6)	.03
**Age (years)**				.31
	<21	56 (11.1)	18 (7.0)	
	21-25	209 (41.4)	117 (45.3)	
	26-30	116 (23.0)	60 (23.3)	
	≥31	124 (24.6)	63 (24.4)	
Single		309 (61.2)	150 (58.1)	.46

^a^Pearson chi-square test with Yates continuity correction.

It felt like I was being reminded that I could have been part of the study, but was not allowed.

Participant 212

It was mostly annoying to get a bunch of SMS saying that I was part of the study, which I wasn’t!

Participant 134

I did not understand what the purpose of the messages was. Was I part of the study and had missed something?

Participant 58

A total of 22.5% (58/258) of the respondents stated that these text messages contributed to feelings of participation and served as a reminder of receiving the smoking cessation intervention later. Some participants expressed that just receiving text messages reminding them of the intervention was helpful. But others claimed that there was a risk of becoming tired of waiting.

It was a reminder of smoking...and I started to feel strongly about quitting.

Participant 114

It was very good to be reminded, but when you want help you get tired of waiting.

Participant 77

Regardless of upcoming support, it affected my perception of smoking negatively. I therefore smoked less.

Participant 55

Of the respondents, 31.4% (81/258) stated that the text messages did not matter or that they did not care about the messages.

### Actions Taken When Being Allocated to the Control Group

More than half of the respondents in the control group (158/258, 61.2%) chose to wait to quit smoking until they had access to the intervention and, of those, 72.8% (115/158) experienced receiving text messages during the waiting time as negative.

Among those who chose to continue to smoke, some claimed that they had wanted to quit smoking but that they needed support, and not being given access to the program was a reason to wait.

I feel like I want to quit, but I can’t put my back into it! The text messages would have been a push in the right direction.

Participant 9

Many comments were about trying to stop or reducing smoking after being randomly allocated to the control group, and 29.8% (77/258) of respondents decided to try to quit smoking without support. Some succeeded in quitting smoking and some cut down but had a relapse after a period without cigarettes.

I cut down considerably, even completely stopped for about 1 month.

Participant 121

I didn’t smoke for 4 weeks, then at a party I started again.

Participant 226

I tried but unfortunately it did not work, now I smoke again.

Participant 196

Only 8.9% (23/258) of respondents reported that they used other support during the waiting time, mostly nicotine aids.

### Willingness to Sign Up for Smoking Cessation After the Trial

Of the respondents, 77.5% (200/258) claimed they were still smoking and had signed up, or were thinking about signing up, to the smoking cessation program. The need for support was expressed in different ways, and the reasons for signing up after the trial included not feeling well, having a disease, and needing support to quit.

Please help me, I feel unwell and really want to quit smoking. I have a disease and my symptoms may get better if I do not smoke.

Participant 226

I have cut down my smoking but need help to quit completely.

Participant 150

Among the respondents who claimed they were thinking about signing up, some were concerned about the sign-up procedure and expressed confusion about whether they needed to sign up again or if the first sign-up when entering the program still counted.

...but what, sign up again? I have already signed up once?

Participant 1657

I thought it (the program) would start automatically when it was my turn. I already signed up for the support, but I didn’t have access to it.

Participant 218

I didn’t know I needed to sign up; wasn’t it just to accept participation?

Participant 137

Only 8.1% (21/258) of respondents answered that they had decided not to sign up for the support although they still smoked. A total of 14.3% (37/258) responded that they had quit smoking during the waiting time and were not interested in signing up for the support.

## Discussion

In RCTs, the estimated effect of the intervention under study can only be understood relative to the control setting; thus, the aim of this study was to explore the experience of being allocated to the control group in the NEXit smoking cessation intervention.

### Principal Findings

The main findings of this study are that most of the participants experienced frustration, disappointment, and irritation about being allocated to the control group. Monthly text messages during the waiting period were perceived as poor and pointless, and not being given the intervention at once was misunderstood as being excluded from the trial. Most of the respondents decided to wait to quit smoking until they had access to the intervention. There was high interest in the novel intervention after the trial.

In the NEXit trial, we decided to ask the control group to quit smoking on their own, but also to inform them that the novel intervention would be available to them after the trial was finished. While a waiting list approach reduces ethical dilemmas, it can also create a feeling of missing out or resentment in the control group. Approximately 61% of the respondents in the control group reported that they had negative feelings about having to wait for the support, and a similar proportion of respondents reported that they had decided to continue smoking while waiting for access to the intervention. While the response rate to the questionnaire was low (34%), having negative feelings about having to wait for the support is still a matter of concern that should be addressed in future studies. Previous research showed that individuals who sign up for lifestyle intervention trials have previously tried to change their behavior but were not able to do so using existing means. They then feel disappointed that they are not given access to a new support tool that they believe would help them, regardless of the fact that the intervention under trial has yet to be proven to be effective [15]. Because the control group behaves in a manner that was not initially planned for, this might create a potential bias in the effect measurement of the intervention, one that cannot be estimated using the primary outcome data collected during trials.

To alleviate the feeling among control group participants of being dismissed or left out, we decided that they were to receive messages throughout the trial to remind and thank them for being part of the trial. Nearly 73% of the respondents found these messages to be poor and pointless. Free-text comments indicated that respondents felt that they were reminded about missing out on the intervention. Furthermore, free-text comments revealed that participants perceived being allocated to the control group as not being allowed to be part of the trial. Thus, not being given the intervention was misunderstood as being excluded from the trial. The monthly reminders were supposed to alleviate the feeling of being left out but seem to have become a reminder of just that.

There was high interest in the novel intervention after the trial, with approximately 71% of responders reporting that they still smoked and had signed up for the intervention. It is, however, interesting that participants were confused as to whether signing up for the trial also meant that they would be given the intervention afterward without actively asking for it. Thus, there seems to be a dichotomy between those who thought that they were excluded and those who thought that they were waiting.

The responses to the questionnaire sent to control group participants strengthen a growing body of evidence that suggests that participants in RCTs are not well aware of the trial design [16-19]. The difficulties include explaining to participants that the intervention under trial is yet to be shown to be effective: control group participants in the NEXit trial felt like they were missing out on support, yet it was not known whether the support would be effective at the trial start. Explaining the concept of placebos and allocation to different groups also presents challenges [20,21].

We took several steps to ensure validity of the results and to prevent bias in the selection of the 143 free-text comments. Free-text comments were read by the first and second authors independently many times. Free-text comments were first selected separately, and then compared and discussed among the authors. One author verified the chosen free-text comments and added some comments relevant to the aim of the study. We excluded free-text comments not agreed on by all authors. Authors were of different ages, sexes, and educational backgrounds.

### Limitations

Limitations include the low response rate: only 34% of the control group responded to the questionnaire regarding their experiences. However, most of the respondents reported negative feelings and comments about being allocated to the control group. This implies that, even if those who did not respond were different from those who did, a large part of the control group were dissatisfied with their participation.

### Conclusion

Future studies should more carefully consider not only the control setting, but also how it is presented to the control group, in order to reduce friction and better reflect the control reference of interest. In the NEXit trial, it would have been advisable to present the trial to participants as comparing 2 different interventions: 1 intervention consisting of immediate access to the SMS text messaging support, and 1 intervention consisting of a motivational phase during which participants would be asked to increase their motivation and attempt to quit smoking on their own with the use of existing support tools, and then be given the SMS support. Hypothetically, this would give the control group a feeling of being part of the trial, having been allocated to an intervention, rather than being told that they were on their own. This would also make the control group more homogeneous, not creating a dichotomy between those who believe they are part of the trial and those who do not. Effect sizes should then be interpreted in light of this control setting.

## Abbreviations

NEXit: Nicotine Exit
RCT: randomized controlled trial
SMS: short message service
